# Public attitudes towards genomic data sharing: results from a provincial online survey in Canada

**DOI:** 10.1186/s12910-023-00967-0

**Published:** 2023-10-07

**Authors:** Holly Etchegary, Georgia Darmonkov, Charlene Simmonds, Daryl Pullman, Proton Rahman

**Affiliations:** 1 Faculty of Medicine, Memorial University, St. John’s, NL A1B 3V6 Canada; 2 Research Initiatives and Services, Memorial University, St. John’s, NL A1B 3V6 Canada; 3 Eastern Regional Health Authority, Memorial University and Rheumatologist, St. John’s, NL A1B 3V6 Canada

**Keywords:** Genomic sequencing, Data sharing, Public engagement, Survey

## Abstract

**Background:**

While genomic data sharing can facilitate important health research and discovery benefits, these must be balanced against potential privacy risks and harms to individuals. Understanding public attitudes and perspectives on data sharing is important given these potential risks and to inform genomic research and policy that aligns with public preferences and needs.

**Methods:**

A cross sectional online survey measured attitudes towards genomic data sharing among members of the general public in an Eastern Canadian province.

**Results:**

Results showed a moderate comfort level with sharing genomic data, usually into restricted scientific databases with controlled access. Much lower comfort levels were observed for sharing data into open or publicly accessible databases. While respondents largely approved of sharing genomic data for health research permitted by a research ethics board, many general public members were concerned with who would have access to their data, with higher rates of approval for access from clinical or academic actors, but much more limited approval of access from commercial entities or governments. Prior knowledge about sequencing and about research ethics boards were both related to data sharing attitudes.

**Conclusions:**

With evolving regulations and guidelines for genomics research and data sharing, it is important to consider the perspectives of participants most impacted by these changes. Participant information materials and informed consent documents must be explicit about the safeguards in place to protect genomic data and the policies governing the sharing of data. Increased public awareness of the role of research ethics boards and of the need for genomic data sharing more broadly is also needed.

**Supplementary Information:**

The online version contains supplementary material available at 10.1186/s12910-023-00967-0.

## Background

The promise of personalized medicine relies heavily on the collection and sharing of vast amounts of genomic and clinical data [1, 2]. Large-scale, government-funded genomic medicine initiatives are underway globally [3]. Commercial genomic test offerings continue to flourish [4], with the DNA of millions of citizens now analysed, stored and sometimes shared by direct to consumer (DTC) genetic testing companies [1, 4]. Accessing and mining large genomic data sets is particularly beneficial for the study of human diseases, fostering tangible scientific discoveries and advances in medicine [5, 6]. Together, large clinical, research and commercial genomic initiatives highlight the growth of data intensive research and the value of globally accessible data [7]. 

However, the research and discovery benefits of genomic data sharing must be balanced against the privacy risks of possible re-identification and misuse of genomic data [8]. Disclosure or misuse of genomic data can lead to harms such as stigmatization or abuse and potential discrimination in education, insurance or employment contexts [9, 10]. Understanding public attitudes and perspectives on data sharing is important given these potential risks and to inform genomic research and policy that aligns with public preferences and needs.

## Public attitudes towards data sharing

Concerns about genomic data storage, sharing, privacy, and unauthorized access are common in the literature [11–15]. Despite privacy concerns, a minority of individuals are willing to share their genomic data with unrestricted access on publicly accessible forums [1, 16, 17]. Some of these participants, however, suggest that willingness to share data varies with social position and privilege [1]. 

While some are motivated to allow access to their genomic data [18], this varies by country and the actors who could access data. For example, individuals are more willing to share data with (and trust in) academic or clinical researchers as opposed to governments or the for-profit sector; individual citizens are largely unsupportive of the idea that the use of their genomic data could result in a commercial profit [19–22]. In a large social science study, spanning over 20 countries and 30 000 individuals, Middleton and colleagues recently revealed that willingness to share genomic data was generally low globally, though lower still in countries such as Germany and Japan [22]. Public trust in genomic research and institutions is also a key factor in determining willingness to share genomic data [1, 22, 23]. These global findings support the need for local and culturally appropriate public communication strategies that transparently explain data sharing policies and processes.

In this study, we report on public attitudes towards genomic data sharing as collected via an online survey that formed part of a program of public engagement research [11, 12, 24]. Our efforts were designed to inform the implementation of genomic medicine and research in the provincial healthcare system of Newfoundland and Labrador (NL), Canada, as well as contribute to the literature on public opinion about genomic data sharing.

## Study setting

The Canadian province of NL has a publicly funded healthcare system comprised of four regional health authorities (Eastern, Central, Western and Labrador-Grenfell). Eastern Health is the largest and the site of the Provincial Medical Genetics Program (PMGP). This clinical genetics service provides all genetic counseling and testing arrangements in the province. Until very recently, genome sequencing had been ordered only by geneticists in the PMGP, with samples sent to labs external to the province for analysis. However, the province is moving towards local testing and new research opportunities with the acquisition of a next generation sequencer.

Our team wished to proactively engage with the public to help inform the implementation of genomic medicine and research in the province. From Fall 2018 to Spring 2019, we created a public advisory council [24] and held public town halls [11], in addition to a province-wide survey [12], to better understand public preferences and expectations around many aspects of sequencing. Here, we focus on public preferences towards data sharing to help inform provincial policy and guideline development, but also contribute to the evidence base on factors associated with public preferences for the use and sharing of their genomic data.

## Methods

Ethics approval was received from the Newfoundland and Labrador Health Research Ethics Board (Ref # 2018.221). Respondents provided informed consent by reading the opening consent pages of the survey and ticking a consent box before they could begin. Survey development and administration are described elsewhere [12], but a brief overview follows.

### Survey administration and sample selection

Survey data were collected over two weeks on the online Survey Monkey platform. Purchased Facebook advertising was the primary method of recruitment. Facebook provided the survey link to *all* registered provincial users in week 1, while targeted advertising in the second week was implemented in the smaller, more rural health authorities to help boost responses from these regions. Study information was further shared through University and health authority social media channels, and the networks of the research team.

### Survey development and content

The research team developed the survey for this study, but definitions/explanations (e.g., genome sequencing, incidental findings, data sharing) were modified or taken directly from the literature and genomic websites with public-facing materials^e.g.,18,25–26^. Development occurred over several weeks and iterations, in discussion with the PMGP and the public council on genomics [11]. The final survey comprised vignettes, scaled, open-ended and demographic items (Additional file 1 contains the complete survey instrument). Content areas included: interest in genome sequencing, information needs, attitudes towards features of sequencing, including unexpected findings, with a final section on genome data sharing. Herein, we focus on this latter section.

### Measures

A vignette was used to describe a hypothetical patient Mary and possible scenarios for data sharing in genomics research. Table 1 displays the vignette, modified from McGuire et al. [18]. As in [18], three items followed, measuring respondent opinion on how comfortable they would be with the release of their genomic and clinical data into: (1) restricted scientific databases only; (2) scientific databases, both publicly accessible and restricted; and (3) only those databases for a specific research study they had consented to be part of. Items were measured on a 5 point Likert scale from strongly agree to strongly disagree. Items 1 and 3 were recoded for univariate analyses such that higher scores indicated more comfort with restricted data usage as opposed to open public databases.

**Table 1:** Genomic data sharing vignette

Mary could be asked for permission for her genomic information and her clinical information, to be released into one or more scientific databases. This could help advance medicine and medical research by allowing other researchers to use this information. There are many scientific databases where Mary’s genetic and clinical information could go; some are maintained by Memorial University, some are maintained by the provincial Department of Health, some by international health organizations and some are maintained by private companies. Some of these databases are publicly accessible – meaning anyone can access them; others are restricted, and can only be accessed by approved researchers through an application process.
In genomic research studies, it is usually the case that neither Mary’s name nor any other personally identifying information about her will ever be released. Nobody will be able to know just from looking at a database that the information belongs to Mary. However, because our genetic information is unique to each one of us, there is a small chance that someone could trace the information back to a patient. The risk of this happening is very small, but may grow in the future. This is possible even if genomic data wasn’t shared with other researchers. As technology advances, databases with many patients’ genomic information will become more valuable to scientists, but there may also be new ways of tracing the information back to patients. With restricted databases, researchers who access patients’ genetic and clinical information will have a professional obligation to protect their privacy and maintain their confidentiality.

One item then asked “if you were asked to consider granting access to your genomic and clinical information for research purposes, how important do you think the following would be for you?” Respondents were provided with 7 options (e.g., where the researchers were from, whether they could withdraw data, whether the research was done by a private company, etc.) and asked to rate these from ‘Not at all important’ to ‘Very Important’ (5 point Likert scale where higher scores indicated greater importance of the factor).

Finally, an item measured respondent opinions’ on the acceptability of the use of patients’ genomic data for various purposes (e.g., profit for commercial companies, profit for government, research related only to provincial residents, benefit sharing, etc.). Respondents were advised to choose all that applied.

This was followed by one open-ended item that asked “Do you have any other comments on the use of genomic data by medical researchers?”

### Demographic, knowledge and self-reported clinical/health system variables

Three items measured (1) self-reported history of a genetic condition in the family, (2) whether respondents had ever had genetic counseling, and (3) whether respondents have ever used services from direct to consumer genetic companies. These items were dichotomized to Yes versus No/Unsure. One item measured respondents’ prior awareness of whole genome sequencing (Before today, how much had you heard about whole genome sequencing?) measured on a 4 point scale ranging from I had never heard of it to I had heard a lot, where higher scores indicated greater prior awareness. One item measured whether respondents knew that our province has a Provincial Health Research Ethics Board that oversees all health research in the province, dichotomized to Yes versus No. We also collected data on sex, marital status, number of children, age in years, range of annual household income, rural/urban residence (as defined by Canada Post for the province) and education level.

### Data analysis

SPSS Version 28 was used for analyses. Descriptive analysis included frequencies, means and standard deviations for all survey items related to data sharing. Univariate analyses used T-tests, ANOVAs or Pearson’s correlations to test the relationships between demographic, knowledge and self-reported clinical variables with the three items measuring comfort with the release of genomic data.

A sample size of around 600 is required for descriptive survey results within +/- 4% points at the 95% confidence interval (https://www.surveysystem.com/sscalc.htm). For univariate analyses, we assumed a medium effect size, power of 0.8, and alpha of 0.05 for significance testing. A sample size of 600 is adequate for univariate inferential analyses such as ANOVAs and correlation [27]. 

### Sample size

The open-ended attitude item was analysed using constant comparison and qualitative description [28, 29]. Answers to the open ended item were placed in a Microsoft Word table where respondent comments were first read and re-read independently by two team members (GD, HE) to begin identifying emerging categories in the language of participants [29]. While a formal codebook was not developed, discussion between the analysts following the coding of the first 30 open comments revealed very similar codes emerging. Once independent coding of all 155 responses was complete, investigators met again to discuss coding decisions. Interrater reliability statistics were not formally calculated. It was decided that responses appearing less than five times in the data would not be included in the final analysis. Differences in coding tended to be minor wording issues (e.g., ‘concerns about use of genomic data by insurance companies’ vs. ‘worried about who could access genomic data’) and were resolved through discussion.

## Results

### Survey response and missing data

We cannot provide an accurate response rate since the number of individuals who saw the survey link but didn’t participate is not known. The online platform recorded 1028 individuals opened the survey link; 901 respondents answered the first question measuring interest in sequencing [12]. Responses to individual items lessened as the survey continued, with just below 700 answering the final items. The total n for each item and analysis is reported subsequently.

### Respondents

Respondents’ mean age was 45 years (SD 13.9; Range 18–82). Most were female (74.5%) and residing in urban centres (73.7%). Most had one child (Mean 1.3; SD 1.2), and were married with a University degree and annual incomes of >$60 000 (Table 2). Over 40% self-reported a genetic condition in their families, but very few reported seeing a genetic counselor (just over 12%, Table 2). Approximately 13% of this sample had used direct-to-consumer testing services.

**Table 2:** Description of survey respondents

Demographic item (total n)	Category levels	Total N (%)
Sex (694)	Male	172 (24.8)
	Female	517 (74.5)
Highest level of education completed (688)	High school or less	77 (11.2)
	Trade school/College diploma	246 (35.8)
	University, undergraduate	181 (26.3)
	University, graduate degree	184 (26.7)
Annual household income (671)	<$20 000 $20 000 - $40 000 $40 000 - $60 000 >$60 000	44 (6.6) 89 (13.3) 116 (17.3) 422 (62.9)
Marital status (691)	Married	459 (66.4)
	Single	145 (21)
	Divorced, separated, widowed	87 (12.6)
Residence (672)	Urban Rural	495 (73.7) 177 (26.3)
History of genetic condition in family (693)	Yes No Unsure	304 (43.9) 175 (25.3) 214 (30.9)
Have you ever used direct to consumer genetic testing such as 23&me or ancestry.com? (697)	Yes No	93 (13.3) 604 (86.7)
Have you ever had genetic counseling? (695)	Yes No	88 (12.7) 697 (87.3)

### Attitudes towards the release of genomic data

**Fig. 1:**
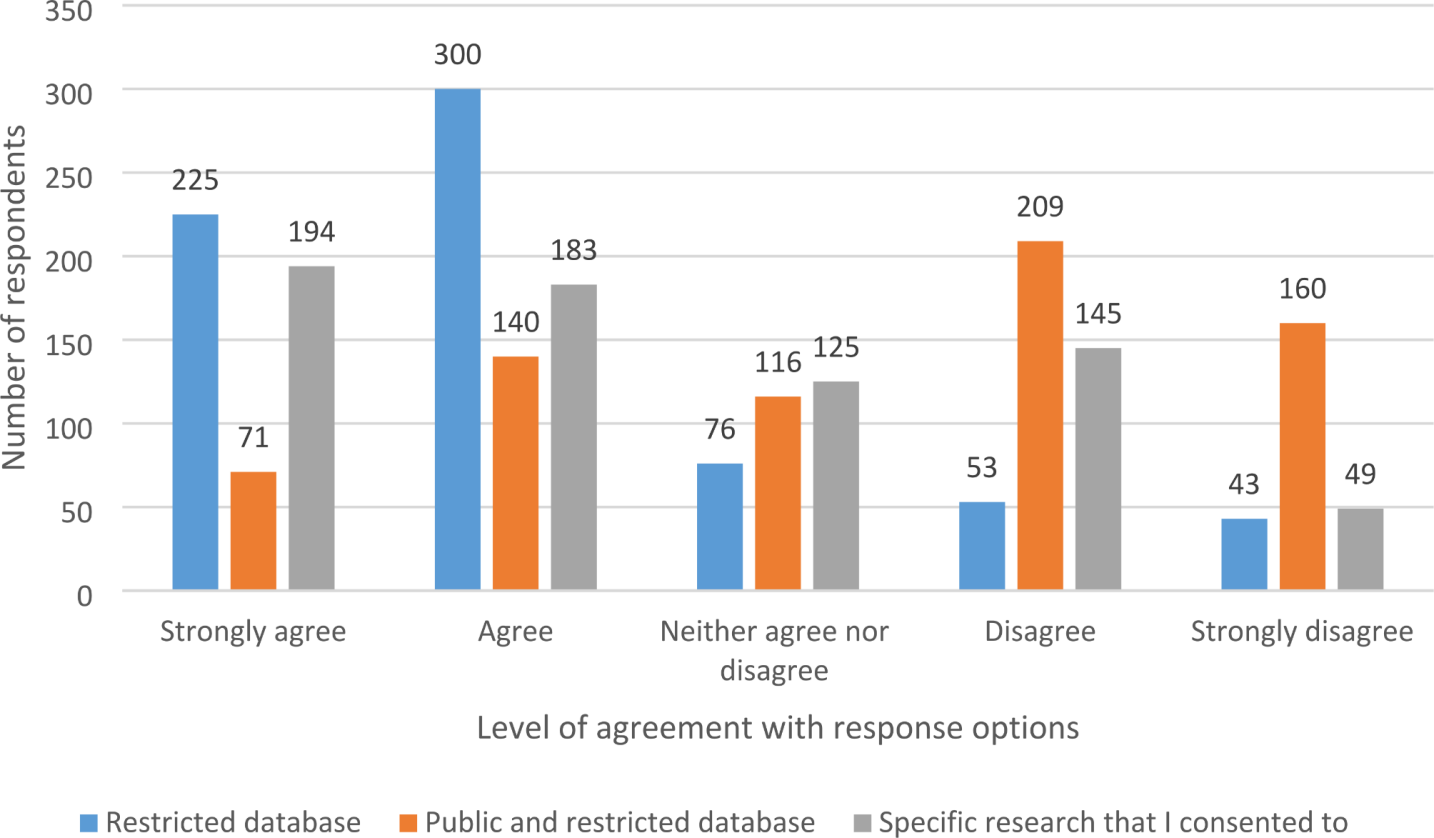
Respondent comfort with the release of their data in different database scenarios

The majority of respondents (total n = 697) either strongly agreed or agreed (525/697; 75%) with the release of their genomic and clinical information into restricted scientific databases only, with smaller percentages disagreeing or having a neutral opinion (Fig. 1).

Respondents who indicated they were *not* aware of the Provincial Health Research Ethics board (HREB) that oversees all research in the province indicated stronger agreement with the release of their data into restricted scientific databases only (X = 4, SD = 1.1) than those who were aware of the jurisdiction’s health research ethics board (X = 3.8, SD = 1.2) [t(690)=-1.9, p < .05]. Prior awareness of whole genome sequencing was also related to this item [F(3, 693) = 2.6, p < .05]. Tukey’s post hoc comparisons showed that those who had heard only a little about sequencing were more comfortable with the release of their data into restricted scientific databases only (X = 3.9, SD = 1) than those who had heard a lot (X = 3.6, SD = 1.4).

Rural/urban residence, sex, age, marital status, number of children, education level, annual household income, prior experience with direct to consumer genetic testing, self-reported genetic condition in the family, and having had genetic counseling were not related to comfort with the release of personal data into restricted scientific databases.

Comparatively, descriptive statistics revealed that most participants (total n = 696) strongly disagreed or disagreed (369/696; 53%) with the release of their information into *both* publicly accessible and restricted databases (Fig. 1).

Previous experience with direct to consumer testing services was significantly related to comfort with the release of data into both publicly accessible and restricted scientific databases. Those who had used such services scored lower on this item (X = 3.1, SD = 1.3) compared to those who hadn’t (X = 3.4, SD = 1.3) [t(689)=-2.4, p < .01]. Recall that this item was not recoded, such that lower means indicate stronger agreement with the release of data into *both* restricted and publicly accessible databases. Sex was also related to this item, such that males were more likely to indicate agreement (X = 3.2, SD = 1.3) than females (X = 3.4, SD = 1.3) [t(680)=-1.7, p < .05].

Age was significantly related to this item. As age increased, so too did disagreement with this item; older respondents disagreed they were comfortable with the release of their data into both publicly accessible and restricted databases [r(678) = 0.47, p < .01].

Knowing the province has a health research ethics board, prior awareness of whole genome sequencing, rural/urban residence, number of children, marital status, education level, household income, self-reported genetic condition in the family, or having had genetic counseling were not related to comfort with the release of data into both publicly accessible and restricted databases.

Finally, Fig. 1 shows that 54% (377/696) strongly agreed or agreed (total n = 696) that they would be comfortable with the release of their genomic information solely for the research project for which they provided consent. Just over 20% disagreed with this statement, with another 18% strongly disagreeing.

This final item measured respondents’ comfort with the release of their data only into databases related to a specific research study for which they provided informed consent. Rural respondents scored higher on this item (X = 2.7, SD = 1.1) than urban respondents (X = 2.4, SD = 1.3) [t(665)= -3.1, p < .05]. Sex was also significantly related to this item such that males were less likely to agree with the release of their genomic and clinical data into databases related specifically to a study they had consented to take part in (X = 3.3, SD = 1.3) than females (X = 3.6, SD = 1.2) [t(681) = 1.9, p < .05]. Knowledge that the province has a health research ethics board (HREB) was also related to this item. Respondents who indicated they were unaware of the HREB scored higher on this item (X = 3.6, SD = 1.2) than those who were aware (X = 3.3, SD = 1.3) [t(689) = 2.3, p < .05], indicating those with knowledge of the ethics board were less likely to agree with releasing their data only into databases related to studies they had consented to be part of.

Having children and age were both positively related to this item. Specifically, as age and number of children increased, so did agreement with the release of personal data into only those databases related to a specific study for which respondents had provided consent [r(679) = 0.17, p < .01 and r(677) = 0.16, p < .01, respectively].

Finally, prior awareness of whole genome sequencing was also related to this item [F(3, 692) = 4.1, p < .05]. Tukey’s post hoc comparisons showed that those who had never heard of sequencing were more comfortable with the release of their data into restricted scientific databases only (X = 3.9, SD = 1.1) than those who had heard a lot (X = 3.3, SD = 1.2).

Self-reported genetic condition in the family, having had genetic counseling, having previously used direct to consumer genetic testing services, education level, annual household income and marital status were not related to this item.

#### Factors important in respondents’ decisions to share genomic data

Respondents were asked to rate a series of items with response options from most important to least important. Table 3 displays the descriptive analysis of factors influencing opinion on the release of genomic data. Considering the endpoints of the scale, almost equal numbers of respondents indicated that where researchers were from was either not important at all (32.3%) or very important (27.8%). It was very important for the majority of respondents to have the ability to withdraw their data at any time (54.1%). A strong majority (84.1%) indicated it was very important for the research to have ethics approval and oversight, and 68.7% said it was very important for the research to have an ethics or privacy officer in place. When asked about whether profits being made on their samples was important, 57.4% indicated that it was, and 45.1% stated that if the research was being done by a private company, this would be an important consideration for them. Finally, 70.5% of participants indicated it was important to know if their sample could be traced back to them.

**Table 3:** Importance of various modifiable factors for the release of genomic data

Item	Not at all Important (1) (%)	2 (%)	3 (%)	4 (%)	Very Important (5) (%)	Mean	SD
**Considerations for granting access to genomic and clinical information for research purposes**							
Where the researchers were from (n = 697)	32.3	10	16.4	13.5	27.8	2.9	1.6
Whether I had the ability to withdraw my data at any time (n = 695)	7.8	5.5	14.1	18.6	54.1	4.1	1.3
Whether the research had ethics approval and oversight (n = 697)	1.4	0.6	3.7	9.8	84.5	4.8	0.7
Whether the research was done by a private company (n = 692)	10.8	4.5	21.4	18.2	45.1	3.8	1.3
Whether the research had an ethics or privacy officer in place (n = 697)	1.4	1.9	9.2	18.8	68.7	4.5	0.9
Whether profits could be made from research using my sample (n = 695)	7.8	4.7	16.1	14	57.4	4.1	1.3
Whether my sample could be traced back to me (696)	5.6	1.7	9.6	12.5	70.5	4.4	1.1

#### Attitudes towards medical researchers’ use of genomic data

**Fig. 2:**
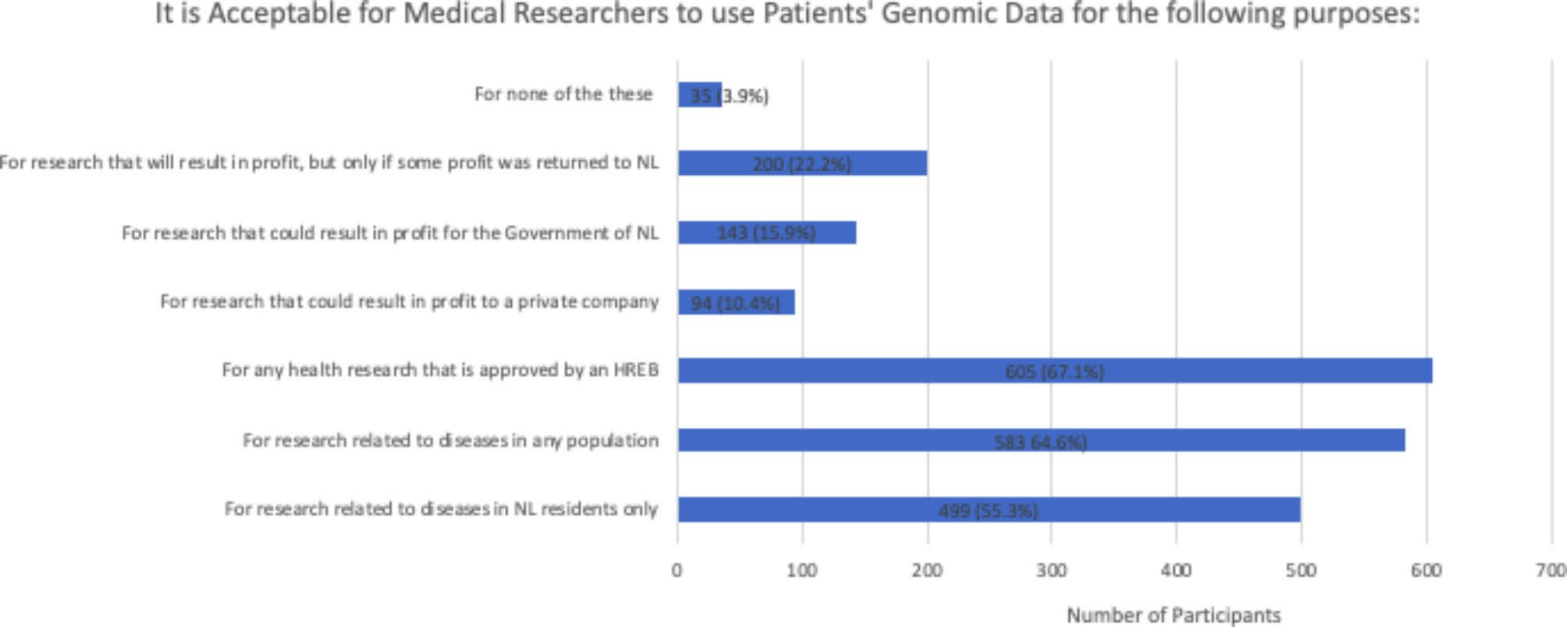
Acceptable uses of genomic data

When asked about medical researchers’ potential uses of their genomic data, 605 respondents (67.1%) indicated they would be comfortable with information being used for any health research study approved by a health research ethics board. A similar percentage (64.6%) indicated their agreement with data being used for research related to disease in any population, while a smaller number (55.3%) indicated support for the use of their data in research in their own jurisdiction. Much smaller percentages agreed with the use of their data for profit, whether that be private companies, the provincial government, or in the context of benefit sharing with some profit being returned to the province (Fig. 2).

### Open ended item

Respondents were given the option of providing additional comments on the use of genomic data by medical researchers in an open item; 155 unique comments were available for analysis. Ideas/themes that appeared five or less times were not included in the final analysis, leaving 104 unique comments categorized into themes. The most prevalent theme in open comments related to profiting from the usage of genomic data. In line with the closed items, open comments revealed strong disagreement with genomic data being used for profit. Specifically, it was discouraged 39 times, including comments that “it should never be for profit” or “if it was going to be for profit, I would want to know more so then I could decide.” A slightly different version of the profit idea, related to benefit sharing, was mentioned 12 times, with comments such as “if drug companies can profit, this should offset treatment costs” or “profit should be shared with those who contributed.”

The next most common theme was related to consent for the usage of genomic data and specifically re-consenting for each different use of the collected data. This appeared in respondents’ comments 22 times, and included phrases such as “signing new agreements every few years.” Storage/usage/sharing of data was also mentioned 15 times, with the vast majority of concerns stemming from insurance companies or employers accessing information. Participants felt that genomic data was important for the advancement of healthcare and noted this in comments 8 times, and finally, some participants commented that they would be ok with sharing their genomic data for any use as long as it was completely anonymous and could not be traced back to them (mentioned 8 times).

## Discussion

As genomic testing becomes integrated into clinical care and large, global genomics research projects continue, data sharing will be essential to reap the full clinical benefits of genomic medicine [30]. Funders, journals and professional associations increasingly endorse data as a public good, with growing requirements from funders to make genomic data publicly available [31]. In light of this trend, international bodies such as the Global Alliance for Genomics and Health (GA4GH) use a human rights framework to frame policy and develop standards to enable responsible genomic data sharing [32]. Thus, the issue of genomic data sharing is timely. In this paper, we described public attitudes towards (and comfort with) the practice.

Results showed a generally moderate comfort level with sharing genomic data, although typically into restricted scientific databases with controlled access. Much lower comfort levels were observed for sharing data into open or publicly accessible databases. These findings are generally consistent with the literature, which shows only a small subset of individuals willing to make their data publicly available [17, 22, 23]. An early trial upon which our data sharing vignette was based [18] revealed 53% of participants opted for public data sharing, higher than our sample. However, a significant minority (47%) in that study chose a more restricted data sharing option. These public preferences are important. Complying with broad data sharing policies, as required by more and more funders, can make it challenging for researchers and institutions to respect the desires of study participants who prefer more restrictive access to their data, and could even result in certain individuals or groups choosing not to participate in research [33]. 

Our findings and others suggest that many general public members are concerned with who will have access to their data [11, 13, 15, 34], with research showing higher rates of approval for access from clinical or academic actors, but much more limited approval of access from commercial entities or governments [22, 33–35]. 

The preference for more restricted access to genomic data and opinions on acceptable uses (and users) have implications for how consent policies for the use of health data are defined and presented to individuals. A recurring theme in our open-ended survey data was the idea of re-consent for each subsequent use of collected data, a preference reported in prior work of our group [36], as well as other studies and reviews [37, 38]. Such onerous consenting practices would no doubt curtail much valuable research. Hence the need to establish culturally appropriate and robust data collection, storage, and sharing practices and governance, and to educate the public on the same if we are to reap maximum benefits from the evolving genomics era in medicine.

It is interesting, however, that when asked specifically about medical researcher’s use of genomic data, 67% of respondents indicated that they would be comfortable with their information being used for any study provided it had approval by a health research ethics board. Theoretically, an ethics-approved study could still release information into a publicly accessible database (and in the main, participants did not agree with sharing their data this way). Participants also noted that whether their sample could be traced back to them would be an important consideration in decisions about data sharing (mean 4.4/5), consistent with a recent, global public opinion survey [22] and also a theme in our open-ended data.

These findings highlight how important it is to better appreciate how research participants understand concepts such as broad consent, re-consent, anonymity of samples and the ability to withdraw samples.

(or not). Transparent and consistent institutional policies will be needed to ensure that researchers and research ethics boards understand what level of data protection can be truthfully promised to study participants and that participants can make data sharing decisions in an informed manner.

Our findings also highlight factors associated with data sharing comfort. Knowledge of the provincial health research ethics board (REB) and prior awareness of genome sequencing were both related such that lack of knowledge and awareness translated into less comfort with open data sharing. These findings highlight the importance of building public knowledge about the research process, including the role and functions of REBs, as well as genomic research more broadly. While we did not ask about data access committees (DACs) in the current survey, they have been proposed as a potential mechanism for promoting the potential benefits of data sharing, while mitigating potential harms [39]. Rather than making data publicly accessible without restrictions, DACs operate under a controlled access environment and are responsible for reviewing data access requests [40]. In our province, DACs associated with a research project would require approval by the provincial REB. In addition to increasing knowledge and awareness about REBs, it could also be useful to build public awareness about DACs.

Findings also indicated men were more comfortable with open data sharing as were prior users of direct to consumer testing services, while rural respondents and older respondents were less comfortable. These findings may be useful for anticipating recruitment where open data sharing is planned and for targeting genomic research recruitment where additional information or education may be necessary.

Ultimately, public trust and willingness to share their data is needed to advance genomic discoveries [22, 23]. Raza and Hall [30] suggest that a greater public understanding of why genomic data sharing is important and improved public trust in the organizations, processes and people involved is going to be critical to realizing the clinical benefits of genomic medicine. Our findings support this suggestion.

Canadians are concerned about the privacy of their health information [41]; similarly, our participants expressed concerns over the use of their data and who had access to it. However, they readily recognized the potential benefits of data sharing and expressed altruistic attitudes about helping others through research, similar to other Canadian samples [34, 36, 42, 43] and globally [14, 15, 22]. Our respondents also indicated their approval of using their genomic data for health research approved by a research ethics board. In the context of rapidly evolving regulations and guidelines for genomics research and data sharing [31, 32], we suggest it is more important than ever to better understand – and consider – the perspectives of participants who will be impacted by these changes. Genomic researchers need to consider participant preferences in study design and consent procedures in order to foster trust and greater research participation. Practically, this means that any participant information material and informed consent documents must be explicit about the safeguards in place to protect genomic data and the policies governing the sharing of their data.

In Canada, a pan-Canadian Human Genome Library (CHGL) is to be launched in 2023, a federated data network for the sharing of locally held genomic and clinical information, but also the point of contact for Canada’s participation in international large-scale genomics projects [47]. In preparation, a core set of informed consent elements have been developed that include not only a description of the data collected and relatively standard consent elements, but also sections on international data sharing, commercial use and future research use [47]. Our findings suggest that participant preferences can be better considered and respected by the inclusion of these latter elements in particular. This guidance and sample consent language [47] should be useful for researchers to enable responsible genomic data sharing, while allowing participants to make informed data sharing decisions.

Study findings must be interpreted in light of limitations. The sample was largely White, well educated, with middle to upper education levels. Findings may not translate to other ethnic groups or individuals of lower income or education levels, nor to specific populations beyond the general public (e.g., patients with cancer). The population was recruited largely through social media, specifically Facebook. While a majority of Atlantic Canadians use Facebook regularly [48], recruitment strategies missed those individuals who are not online users; our recruitment strategy also does not allow us to compare survey respondents with non-respondents.

More broadly, findings must be interpreted in the context of how the survey defined and presented data sharing concepts and terms. It is unknown if all survey items were sufficiently clear and unambiguous to respondents, which could have impacted responses. For example, we did not define ‘where the researchers were from’ so we cannot know if respondents interpreted this item as researchers from outside the province, country or local academic institution. Our definition of ‘publicly accessible’ databases and the coupling of this concept with restricted databases in one survey item could have skewed responses towards disagree. It is possible comfort with the sharing of data into restricted databases would have been higher had those concepts been disentangled and measured in two survey items. Similarly, while our findings suggest respondents are less supportive of the use of their data for profit, the survey did not distinguish between profit in terms of data being used for commercial use versus scientific research that eventually could lead to commercialization. Nor did the survey define ‘private company.’ However, respondents did not strongly endorse the sharing of their data with these actors, whatever way ‘private company’ was understood by them and this is generally consistent with the literature. Nonetheless, attitudes about such nuances of profit and who exactly is using data for profit were not captured in the survey and could have been confusing for respondents. While we worked with our 12-member public advisory council to create and revise the survey and followed their advice to present items as simply as possible, we cannot know exactly how survey respondents interpreted these concepts, a necessary limitations of surveys.

## Conclusions

Newfoundland and Labrador, Canada has long been recognized as an important genetic isolate [49] such that there has been a history of interest in exploiting this genetic data for both its potential health benefits, but also for commercial gain. As such we were curious as to how the general public perceived the importance of maintaining control of access to their genetic data. We believe our findings are relevant not only to this population but will contribute to the literature on public opinion about genomic data sharing more generally.

Globally, there is growing recognition of the value of patient and public engagement for improving healthcare, research and public health policy decisions [44, 45]. From a public health and human rights perspective, everyone has the right to benefit from advances in medicine, including genomics. But this can only ethically happen if there is opportunity for individuals to share their views in ways that might inform policy, practice and research in genomics.

Public engagement is critical to ensure researchers and others understand the perspectives of participants who are asked to share their genomic data. Participant education materials and informed consent documents for genomic research must be explicit about the safeguards in place to protect genomic data and the local policies governing data sharing. Increased general public awareness of the role and function of health research ethics boards could help engender public trust in genomic research initiatives.

### Electronic supplementary material

Below is the link to the electronic supplementary material.

Additional file 1: Survey instrument

## Data Availability

The datasets used and/or analysed during the current study are available from the corresponding author on reasonable request.

## List of abbreviations

CHGL: Canadian Human Genome Library
DTC: Direct to Consumer
GA4GH: Global Alliance for Genomics and Health
NL: Newfoundland and Labrador
PMGP: Provincial Medical Genetics Program
REB: Research Ethics Board

## References

[1] HaeuSermann T, Fadda M, Blasimme A, Tzovaras B, Vay E (2018). Data sharing and the social gradient of genomic privacy. AJOB Emp Bioeth.

[2] Kaye J (2012). The tension between data sharing and the protection of privacy in genomics research. Ann Rev Genomics Hum Genet.

[3] Stark Z, Dolman L, Manolio TA, Ozenberger B, Hill SL, Caulfied MJ (2019). Integrating genomics into healthcare: a global responsibility. Am J Hum Genet.

[4] Hogarth S, Saukko P (2017). A market in the making: the past, present and future of direct-to-consumer genomics. New Genet Soc.

[5] Vayena E, Blasimme A (2018). Health research with big data: time for systemic Oversight. J Law Med & Ethics.

[6] Ball M, Bobe J, Chou M, Clegg T, Estep P, Lunshof J (2014). Harvard personal genome project: Lessons from participatory public research. Genome Med.

[7] Shabani M, Knoppers B, Borry P (2015). From the principles of genomic data sharing to the practices of data access committees. EMBO Mol Med.

[8] Gymrek M, McGuire A, Golan D, Halperin E, Erlich Y (2013). Identifying personal genomes by surname inference. Science.

[9] Annas G, Elias S. Genetic privacy and DNA databanks. In: Annas G, Elias S, Editors. Genomic messages. How the evolving science of genetics affects our health, families, and future. New York: HarperCollins;191–215.

[10] McEwen JE, Boyer JT, Sun KY (2013). Evolving approaches to the ethical management of genomic data. Trends Genet.

[11] Etchegary H, Winsor M, Power A, Simmonds C (2021). Public engagement with genomic medicine: a summary of town hall discussions. J Community Genet.

[12] Etchegary H, Pullman D, Simmonds C, Rabie Z, Rahman P (2021). Identifying aspects of public attitudes towards whole genome sequencing to inform the integration of genomics into care. Public Health Genomics.

[13] Metcalfe S, Hickerton C, Savard J, Terrill B, Turbitt E, Gaff C (2018). Australians’ views on personal genomic testing: focus group findings from the Genioz study. Eur J Hum Genet.

[14] American Society of Human Genetics. Public attitudes toward genetics & genomics research: literature and polling review report. 2020. https://www.ashg.org/wp-content/uploads/2020/01/2020-Public-Views-Genetics-Literature-Review.pdf. Accessed 17 Jan 2023.

[15] Genetic Alliance UK. Genome sequencing: What do patients think? Patient Charter. 2015. https://www.geneticalliance.org.uk/media/1924/patientcharter-genome-sequencing-what-do-patients-think.pdf. Accessed 17 Jan 2023.

[16] Vayena E, Mastroianni A, Kahn J (2012). Ethical issues in health research with novel online sources. Am J Public Health.

[17] HaeuSermann T, Greshake B, Blasimme A, Irdam D, Richards M, Vayena E (2017). Open sharing of genomic data: who does it and why?. PLoS ONE.

[18] McGuire A, Oliver J, Slashinski M, Gravey J, Wang T, Kelly P (2011). To share or not to share: a randomized trial of consent for data sharing in Genome Research. Genet Med.

[19] Critchley C, Nicol D, Otlowski M (2015). The impact of commercialisation and genetic data sharing arrangements on public trust and the intention to participate in biobank research. Public Health Genomics.

[20] Gaskell G, Gottweis H, Starkbaum J, Gerber MM, Broerse J, Gottweis U (2013). Publics and biobanks: pan-european diversity and the challenge of responsible innovation. Eur J Hum Genet.

[21] Milne R, Morley KI, Howard H, Niemiec E, Nicol D, Critchley C (2019). Trust in genomic data sharing among members of the general public in the UK, USA, Canada and Australia. Hum Genet.

[22] Middleton A, Milne R, Almarri M, Anwer S, Atutomu J, Baranova E (2020). Global public perceptions of genomic data sharing: what shapes the willingness to donate DNA and health data?. Am J Hum Genet.

[23] Broekstra R, Maeckelberghe M, Aris-Maijer J, Stolk R, Otten S. Motives of contributing personal data for health research: (non-)participation in a dutch biobank. BMC Med Ethics. 2020; 21(62). 10.1186/s12910-020-00504-3 PMC7382031 32711531

[24] Abhyankar S, Etchegary H, Labrador Public Advisory Council on Genomics (PACG). Rolling out genomic screening: the Newfoundland and. 2019. In: BMJ Partnership in Practice. https://blogs.bmj.com/bmj/2019/12/17/rolling-out-genomic-screening/ Accessed 17 Jan 2023.

[25] Bijlsma R, Wessels H, Wouters R, May A, Ausems M, Voest E (2018). Cancer patients’ intentions towards receiving unsolicited genetic information obtained using next-generation sequencing. Fam Cancer.

[26] Genome England. What is a genome? https://www.genomicsengland.co.uk/the-100000-genomes-project/understanding-genomics/what-is-a-genome/ Accessed 17 Jan 2023.

[27] Cohen J (1992). A power primer. Psych Bull.

[28] Sandelowski M (2010). What’s in a name? Qualitative description revisited. Res Nurs Health.

[29] Glaser B, Straus A (2017). The Discovery of grounded theory: strategies for qualitative research.

[30] Raza S, Hall A (2017). Genomic medicine and data sharing. Br Med J.

[31] Sabatello M, Martschenko D, Cho M, Brothers K (2022). Data sharing and community engaged research. Science.

[32] The Global Alliance for Genomics and Health. http://genomicsandhealth.org/. Accessed 15 Nov 2022.

[33] McGuire AL, Basford M, Dressler L, Fullerton S, Koenig B, Li R (2011). Ethical and practical challenges of sharing data from genome-wide association studies: the eMERGE Consortium experience. Genome Res.

[34] Paprica P, Nunes de Melo M, Schull M (2019). Social licence and the general public’s attitudes toward research based on linked administrative health data: a qualitative study. CMAJ.

[35] Spithoff S, Stockdale J, Rowe R, McPhail B, Persaud N (2022). The commercialization of patient data in Canada: ethics, privacy and policy. CMAJ.

[36] Pullman D, Etchegary H, Gallagher K, Hodgkinson K, Keough M, Morgan D (2012). Personal privacy, public benefits, and biobanks: a conjoint analysis of policy priorities and public perceptions. Genet Med.

[37] Sanderson S, Brothers K, Mercaldo D, Clayton E, Antommaria A, Aufox S (2017). Public attitudes toward consent and data sharing inbiobank research: a large multi-site experimental survey in the US. Hum Genet.

[38] Garrison N, Sathe N, Antommaria A, Holm L, Sanderson S, Smith M (2016). A systematic literature review of individuals perspectives on broad consent and data sharing in the United States. Genet Med.

[39] Cheah P, Piasecki J (2020). Data Access Committees. BMC Med Ethics.

[40] Kaye J, Hawkins N (2014). Data sharing policy design for consortia: challenges for sustainability. Genome Med.

[41] Office of the Privacy Commissioner of Canada. 2020-21 Survey of Canadians on Privacy-Related Issues https://www.priv.gc.ca/en/opc-actions-and-decisions/research/explore-privacy-research/2021/por_2020-21_ca/ Accessed 9 Aug 2023.

[42] Teng J, Bentley C, Burgess M, O’Doherty K, McGrail K. Sharing linked data sets for research: results from a deliberative public engagement event in British Columbia, Canada. Int J Pop Data Sci 2019: 4(1). 10.23889/ijpds.v4i1.1103 PMC8142623 34095532

[43] Genome Canada. Public perceptions of genomics in Canada. 2022. https://genomecanada.ca/how-we-work/genomics-in-society/key-findings-public-perceptions-of-genomics-in-canada-survey-2022/ Accessed 9 Aug 2023.

[44] Lemke A, Harris-Wai J (2015). Stakeholder engagement in policy development: challenges and opportunities for human genomics. Genet Med.

[45] Vanstone M, Canfield C, Evans C, Leslie M, Levasseur M, MacNeil M (2023). Towards conceptualizing patients as partners in health systems: a systematic review and descriptive synthesis. Health Res Policy Sys.

[46] Oliver JM, Slashinski MJ, Wang T, Kelly PA, Hilsenbeck SG, McGuire AL (2012). Balancing the risks and benefits of genomic data sharing: genome research participants’ perspectives. Public Health Genomics.

[47] Longstaff H, Flamenbaum J, Richer E, Egar J, McMaster C, Zawati M (2022). Core elements of participant consent documents for canadian human genomics research and the National Human Genome Library: Guidance for policy. CMAJ.

[48] Statista. Frequency of use of Facebook products and services in Canada, by region. 2018. https://www.statista.com/statistics/822150/canada-facebook-products-services-use-by-region/. Accessed 15 Nov 2022.

[49] Kosseim P, Pullman D, Perrot-Daly A, Hodgkinson K, Street C, Rahman P. (2013) Privacy Protection and Public Good: Building a Genetic Database for Health Research in Newfoundland and Labrador. J Am Med Informatics Ass 2013; 20(1): 38–43. 10.1136/amiajnl-2012-001009 PMC3555321 22859644

