# Supplementary material for: Public attitudes towards genomic data sharing: results from a provincial online survey in Canada
Source: BMC Med Ethics. 2023 Oct 7;24:81. doi: 10.1186/s12910-023-00967-0 (PMC10560413; doi:10.1186/s12910-023-00967-0)
Supplement: Supplementary file 1 — Additional file 1: Survey instrument [file 12910_2023_967_MOESM1_ESM.pdf]

Public opinion survey about whole genome sequencing

INFORMATION AND CONSENT

We are inviting residents of Newfoundland and Labrador aged 18 and older to take part in a public opinion survey about whole genome sequencing.

Please read the following consent agreement to inform you about the survey, then proceed when you are ready.

No special background or knowledge is needed to complete the survey. Taking part in this study is voluntary. It is up to you to decide whether to be in the study or not. You can decide not to take part in the study. If you decide to take part and complete the survey, you are free to stop at any time. Taking part in this survey will not affect any healthcare which you or your family receives.

DESCRIPTION OF STUDY

This survey will assess your opinions about whole genome sequencing. Knowing more about our genomes can help us determine the genetic causes of diseases or give information about a person's future risk of developing disease. It can also help doctors choose the right medications for people. But there may also be privacy concerns about collecting and storing our genomic data. We would like to survey Newfoundlanders and Labradorians to get their opinion on some of these issues.

It takes about 20 minutes to fill out. You can finish it by yourself, or with help from another person, such as a family member or friend. Please answer all questions to the best of your ability; the data collected will be used for scientific research to assess the beliefs and opinions which Newfoundlanders and Labradorians have regarding whole genome sequencing. Please note that there are no right or wrong answers; we are only interested in your opinions. If you come to a question you do not want to answer or do not understand, you can skip it.

Participants will have the option to enroll in a draw for a $100 gift card.

PRIVACY

Protecting your privacy is an important part of this study. Your answers to the following survey will be completely anonymous, and no effort will be made to use the information to identify who you are. The answers you provide us are confidential and will only be reported in aggregate. Your name will not be recorded or used in any papers or reports prepared from the survey data.

You may have found this survey through social media, but no information you provide here is linked to outside profiles (e.g., Facebook account). This survey is administered using Survey Monkey, stored securely and not shared. The on-line survey company hosting this survey, Survey Monkey, is located in the United States. The US Patriot Act allows authorities to access the records of internet service providers. Therefore, anonymity and confidentiality cannot be fully guaranteed if you choose to participate in this survey. However, ONLY the information you provide in this survey will be stored and may be accessed in the US. Your computer IP address or email address will NOT be automatically collected.

LIABILITY STATEMENT

By completing this survey, you are implying that you are consenting to be in this study. Giving us your consent tells us you understand the information about the research study. By consenting to be a participant in this study, you are not giving up your legal rights. Researchers involved in this research study still have their legal and professional responsibilities.

ACCESS TO RECORDS

Since the survey will be completed anonymously, we are unable to provide you access to records of your survey responses once it has been collected.

USE OF YOUR STUDY INFORMATION
The research team will collect and use only the information they need for this research study. This information will include your:
- Interest in whole genome sequencing
- Attitude about the uses of your genomic data
- Opinions about giving consent for the use and storage of your genomic data
- Opinions about unexpected findings that might come up during genome sequencing
- Demographic information, such as age, sex, and household income so we can compare the responses of different groups of people

Collected data will be kept secure by the research team, under the responsibility of Dr. Holly Etchegary, the study principal investigator.

QUESTIONS OR PROBLEMS?
If you have any questions about taking part in this study, you can contact the primary investigator who is in charge of the study at this institution. That person is:

Dr. Holly Etchegary PhD
Primary Investigator
Clinical Epidemiology Unit
Faculty of Medicine, Memorial University
Room 4M210, Medical Building
Email: holly.etchegary@med.mun.ca | Phone: 709-864-6605

FURTHER CONTACT DETAILS:
You can also talk to someone who is not involved with the study at all, but who can advise you on your rights as a participant in any research study. This person can be reached through:

ETHICS OFFICE
Health Research Ethics Authority
709-777-6974 or by email at info@hrea.ca

FUNDING
This study is made possible through a grant provided by the Atlantic Canadian Opportunities Agency (ACOA).

* 1. **Do you consent to participating in this study?**

By selecting **“Yes”** and click **"Next,"** you are consenting to be in this study. It tells us you understand the information about the research study. When you select “yes” and consent to be a participant in this study, you are not giving up your legal rights. Researchers or agencies involved in this research study still have their legal and professional liabilities

- ○ Yes
- ○ No

Public opinion survey about whole genome sequencing

ELIGIBILITY

* 2. Are you 18 years old, or older?

- Yes
- No

* 3. Are you a resident of Newfoundland and Labrador?

- Yes
- No

Public opinion survey about whole genome sequencing

GENETIC INFORMATION

Thank you for giving us your opinions about whole genome sequencing – a new way of looking at our DNA. Your opinions will help us decide how we should present this new test to patients in Newfoundland and Labrador in both a clinical and a research setting.

To help you give your opinion, here is some information about our genome and our health:

Our genome and our health

Being healthy or getting sick are affected by many things. One of these things is our genome. It contains all the instructions for making and maintaining you. It is written in a chemical code called DNA. All living things have a genome - plants, bacteria, viruses and animals.

Your genome is all 3.2 billion letters of your DNA. It contains around 20,000 genes. Genes are the instructions for making the proteins our bodies are built of – from the keratin in hair and fingernails to the antibody proteins that fight infection.

What is DNA?

Our genetic code is written as DNA (deoxyribose nucleic acid). It is a long molecule that is a string of letters: A, T, C and G. Combinations of these letters tell our bodies how to function.

What is genome sequencing?

Sequencing is a technique that is used to 'read' DNA. It finds the order of the letters of DNA (A, T, C and G), one by one. So, sequencing a human genome means finding the sequence of someone's unique 3 billion letters of DNA. This is a new way of looking at DNA, where all of a person's DNA is examined in a special machine and information on all of a patient's DNA will be stored in a special computer file.

Think of books in a library. The whole genome sequencing approach is like having a printout that presents every single character in the text of every single book in a library, in the same order as the books were on the shelf.

Why sequence a genome?

Learning more about genomes can help us to determine the genetic cause for many diseases and disorders. It can also give information about a person's risk of developing diseases in the future. It can give parents information about whether they carry variations in their genomes that could cause disease in their children (we call this carrier testing, such as for Cystic Fibrosis). Genome sequencing could also be used to help doctors choose medications for patients.

GENETIC TESTING

The patient - Mary

Let's consider an imaginary patient, Mary. Mary has several health problems, including diabetes and a heart valve problem. Today, she is seeing her heart specialist to talk about the possibility of needing her heart valve replaced someday. He thinks that will be necessary within five years, but if she gets sick suddenly, it could happen sooner. After her heart surgery, he tells her she will need to take a blood thinner. The heart doctor explains that he used to start all his patients on the same dose of blood thinner, and through trial and error over a few months, he would finally get the dose right. Now, however, he can do a test using whole genome sequencing that will predict how Mary's body will handle the blood thinner. He explains this will help him figure out the right dose for her faster. He would like to do the genetic test today and record it in her medical record, so the information will be available to him when she needs it.

4. In this situation, do you think Mary should get the genetic test

- ◯ Yes
- ◯ No
- ◯ Not sure

5. The lab machine that does Mary's genetic test can do one test or 100 genetic tests at the same time. All these tests look for changes that affect the way people respond to drugs. Doing all these tests costs the same and goes almost as fast as doing one. Do you think Mary and her doctor should go ahead and do other tests to find out how her body will react to other medications she might need in the future?

- ◯ Yes
- ◯ No
- ◯ Not sure

6. Some genes can make certain drugs work better or worse, while other genes, while rare, can cause your body to have very unpleasant reactions to certain drugs. Knowing this, do you think **you personally** would be interested in having your genome sequenced to find variations that could affect which drugs are prescribed for you?

- ◯ Yes
- ◯ No
- ◯ Not sure

7. Is there anything you would like to comment on about the use of whole genome sequencing for helping doctors make choices about drugs that are prescribed to their patients?

[ ]

Public opinion survey about whole genome sequencing

PATIENT CONSENT

The patient - Mary

There are several ways doctors get permission from patients to do things. Everyone who gets medical treatment in the province signs a form at least once agreeing to their routine treatment (like bloodwork). For other things that are common, doctors will often ask. For example, they might say, “I’d like to get an x-ray of your hand, is that ok?” If things are risky or different, they might have patients sign an extra form. This happens most often before we have surgery.

8. If Mary signed only a routine treatment form, do you think that by signing that form, Mary gave permission for her doctor to do the genomic test for the blood thinner?

- ◯ Yes
- ◯ No
- ◯ Not sure

9. Do you think this means she gave permission to do other genomic tests for different medications, even ones the doctor does not plan to prescribe right now?

- ◯ Yes
- ◯ No
- ◯ Not sure

Public opinion survey about whole genome sequencing

INCIDENTAL FINDINGS

We have been talking about one potential use of whole genome sequencing in Mary's clinical care. Whether genome sequencing is used for a clinical purpose (such as choosing the best medications for patients) or a specific research purpose (such as looking for the cause of a rare disease), it is important to have a plan for managing other kinds of results, known as secondary or incidental findings. These are defined as results not related to the primary reason for testing, but which are discovered "incidentally" in the process of looking for other things.

The patient - Mary

For example, we know Mary has a heart valve problem. If she has the genetic test for blood thinner, but the lab finds a genetic change that means she is at high risk for getting bowel cancer someday, should they report this incidental finding to Mary and her doctor? Many people might say yes, because Mary's healthcare might be changed now that the variant for cancer has been found. But what if an unexpected variant was found that suggested Mary was at high risk for developing Alzheimer's disease someday? This finding might predict what will happen to Mary someday, but there are no preventive measures that could be taken and Mary might not want to know about this variant. It is important to remember that testing positive for a particular predictive variant (like a cancer gene, or an Alzheimer gene) does not mean Mary WILL get that disease, it only means she has a higher chance of getting the disease than the normal population.

Today, there are no clear guidelines for the disclosure or non-disclosure of incidental findings resulting from whole genome sequencing. Our research team will need to develop local policies about unexpected findings and we would like to hear from patients and members of the public on this important issue.

10. If you were in Mary's position, how interested would you be in receiving ALL whole genome sequencing results, even if they were unexpected and not related to the main reason you had testing?

Definitely interested	Somewhat interested	Not sure	Not interested	Definitely not interested
◯	◯	◯	◯	◯

INCIDENTAL FINDINGS

There are four kinds of unexpected, incidental findings that might be found as a result of having your genome sequenced.

Category 1
A gene variant that predisposes you to a disease that can be prevented or treated.
Example: You have a gene variant which means you are much more likely to develop colon cancer. In this case, we may recommend that you have earlier and more frequent screening (e.g., colonoscopy) than the general population.

Category 2
A gene variant that predisposes you to a disease that cannot be prevented or for which no current effective treatment has been established yet.
Example: You have a gene variant which suggests you are more likely to develop Alzheimer's disease. Alzheimer's disease cannot be treated or prevented.

Category 3
A gene variant that does not affect your health, but may be important to the health of your other relatives, such as your children or future children.
Example: You could learn that you have a variant in the gene that may cause Cystic Fibrosis in future children if your partner also had this variant. This means you are a carrier for that condition, but will never be affected by it yourself.

Category 4
Uncertain gene variants, meaning they may or may not be important to your health or the health of your relatives.
Example: You have a so-called unclassified variant, which implies you do have a variant, for example, for an increased risk of bowel cancer, but the significance is currently unknown.

11. Please tell us your interest in receiving each type of unexpected findings:

	Definitely interested	Somewhat interested	Not sure	Not interested	Definitely not interested
Category 1 – preventable or treatable disorders	○	○	○	○	○
Category 2 – disorders that are currently not preventable nor treatable	○	○	○	○	○
Category 3 – carrier results that may have implications for other family members, but not your health	○	○	○	○	○
Category 4 – results that are currently unclassified, meaning we do not yet know exactly what impact they will (or will not) have on your health or future health	○	○	○	○	○

12. The following items cover other features of unexpected results. Please indicate how strongly you agree or disagree with the following statements:

	Strongly agree	Agree	Neither agree nor disagree	Disagree	Strongly Disagree
Patients should be told about all possible unexpected results and be given a choice about which ones they want returned before having their genome sequenced.	○	○	○	○	○
I would be comfortable having all unexpected results found during sequencing being placed in my medical record.	○	○	○	○	○
Even if nothing can be done about them yet, recording all unexpected results that are found during sequencing in a patient's medical record is important in case more is found about what the result means in the future.	○	○	○	○	○
I would be comfortable with unexpected results being shared with my family doctor.	○	○	○	○	○
I would expect someone to follow up with me at a later time if new information is found about the unexpected results.	○	○	○	○	○

13. Is there anything else you would like to add about unexpected results found during whole genome sequencing?

INFORMATION YOU WOULD WANT

14. Overall, how interested would you be in getting your whole genome sequenced?

Definitely interested	Somewhat interested	Not sure	Not interested	Definitely not interested
○	○	○	○	○

15. Before today, how much had you heard about whole genome sequencing?

I had never heard about it	I had heard a little	I had heard a fair amount about it	I had heard a lot
○	○	○	○

16. I would be interested in having my genome sequenced if it could help me learn about my risks for treatable diseases.

Definitely interested	Somewhat interested	Not sure	Not interested	Definitely not interested
○	○	○	○	○

17. If you were ever in the situation to consider having your genome sequenced, how important do you think the following information would be for you?

	Not important at all				Very important
Information about the kinds of results possible	○	○	○	○	○
Whether my sample would be used in future research studies	○	○	○	○	○
Whether the results have implications for my family	○	○	○	○	○
Whether I could download my raw genetic data	○	○	○	○	○
Information about who is responsible for protecting my sample	○	○	○	○	○
Whether a disease was treatable or not	○	○	○	○	○
How results would be reported to me	○	○	○	○	○
Support available to help me interpret the results	○	○	○	○	○
Who could have access to my genomic information	○	○	○	○	○

18. Please tell us if there any other information you would want to know about?

Public opinion survey about whole genome sequencing

CONCERNS

19. The following items ask for your opinion about some features of whole genome sequencing. Please indicate how strongly you agree or disagree with the following statements:

	Strongly Agree	Agree	Neither agree nor disagree	Disagree	Strongly disagree
I would be concerned that, if the results of a genomic test revealed something unexpected, it would cause me extra worry.	○	○	○	○	○
I would be concerned that a genomic test would cause problems with insurance.	○	○	○	○	○
Before having my genome sequenced, I would want some control over who I allowed to access my sequencing results.	○	○	○	○	○
I would allow my pharmacist to have access to my genomic test results to help make decisions about medications prescribed for me.	○	○	○	○	○
A person should have genetic counseling before deciding to have their genome sequenced.	○	○	○	○	○
I would be concerned that a genomic test would cause problems with employment.	○	○	○	○	○
It is important my healthcare provider (family doctor or specialist) tell me about any genomic tests before they are done.	○	○	○	○	○
I would want my family doctor to have information about my sequencing results up to inform decisions about my care.	○	○	○	○	○
If I had my genome sequenced as part of a research study, I would want the ability to withdraw my genomic sample from the study if I later changed my mind.	○	○	○	○	○

USE OF DATA

20. The following items ask for your opinion about some features of whole genome sequencing. Please indicate how strongly you agree or disagree with the following statements:

	Strongly Agree	Agree	Neither agree nor disagree	Disagree	Strongly disagree
I would have my genome sequenced if it could help me learn about my risks for future diseases that are not currently treatable.	○	○	○	○	○
If I had my genome sequenced, I would share my results with other family members who might also be affected.	○	○	○	○	○
If I had my genome sequenced, I would prefer to sign a written consent form about how my sample could be used.	○	○	○	○	○
I would only have my genome sequenced if I could choose upfront how my data could be used (e.g., for my healthcare, for a research project, for both, for some other purpose).	○	○	○	○	○
In general, we worry too much about privacy and genetics	○	○	○	○	○
I trust that my genomic information would be safe and secure if it were to be stored in my medical records.	○	○	○	○	○

21. It is generally acceptable for medical researchers to use patients' genomic data for the following purposes (tick all that apply):

- ☐ For research related to diseases in Newfoundland and Labrador residents only
- ☐ For research related to diseases in any population
- ☐ For any health research that is approved by a health research ethics board
- ☐ For research that could result in profit to a private company (e.g., drug manufacturers or private sequencing companies)
- ☐ For research that could result in profit for the provincial government (e.g., researchers apply for anonymous genomic samples and pay a price for them)
- ☐ For research that will result in profit, but only if some of that profit was returned to the province of Newfoundland and Labrador
- ☐ For none of these

22. Do you have any other comments on the use of genomic data by medical researchers?

[ ]

Public opinion survey about whole genome sequencing

SECONDARY USE OF DATA

Genomic research needs big samples. With participants' permission, researchers around the world are sharing genomic data for use in medical research. Genomic sequence data is most valuable when it is linked to information about a patient's medical history (clinical information). We are interested in your opinion about storing and using genomic information for medical research.

The patient - Mary

Mary, while having the genetic test for blood thinner, could have been asked by her doctor if her sequence data could be stored and used, not only for her clinical care, but also for future research.

In this case, Mary could be asked for permission for her genetic information, and/or her clinical information, to be released into one or more scientific databases. This could help advance medicine and medical research by allowing other researchers to use this information. There are many scientific databases where Mary's genetic and clinical information could go; some are maintained by Memorial University, some are maintained by the provincial Department of Health, some by international health organizations and some are maintained by private companies. Some of these databases are publicly accessible; others are restricted, and can only be accessed by approved researchers through an application process.

In genomic research studies, it is usually the case that neither Mary's name nor any other personally identifying information about her will ever be released. Nobody will be able to know just from looking at a database that the information belongs to Mary. However, because our genetic information is unique to each one of us, there is a small chance that someone could trace the information back to a patient. The risk of this happening is very small, but may grow in the future. This is possible even if genomic data wasn't shared with other researchers. As technology advances, databases with many patients' genomic information will become more valuable to scientists, but there may also be new ways of tracing the information back to patients. With restricted databases, researchers who access patients' genetic and clinical information will have a professional obligation to protect their privacy and maintain their confidentiality.

The decision of whether or not to allow genetic and clinical information about Mary to be released into scientific databases for research is completely up to Mary.

23. If you were in Mary's situation and asked about the release of your genomic and clinical information, please indicate how strongly you agree or disagree with the following statements:

	Strongly agree	Agree	Neither agree nor disagree	Disagree	Strongly Disagree
I would be comfortable with the release of my genetic and clinical information into restricted scientific databases only.	○	○	○	○	○
I would be comfortable with the release of my genetic and clinical information into scientific databases, both publicly accessible and restricted.	○	○	○	○	○
I would not be comfortable with the release of my genetic and clinical information into any scientific database, except those used and maintained for a specific research study that I agreed to take part in.	○	○	○	○	○

SECONDARY USE OF DATA

24. If you were asked to consider granting access to your genomic and clinical information for research purposes, how important do you think the following would be for you?

	Not important at all				Very important
Where the researchers were from	○	○	○	○	○
Whether I had the ability to withdraw my data at any time	○	○	○	○	○
Whether the research had ethics approval and oversight	○	○	○	○	○
Whether the research was done by a private company	○	○	○	○	○
Whether the research had an ethics or privacy officer in place	○	○	○	○	○
Whether profits could be made from research using my sample	○	○	○	○	○
Whether my sample could be traced back to me	○	○	○	○	○

25. Is there anything else you would like to add about unexpected results found during whole genome sequencing?

Public opinion survey about whole genome sequencing

GENERAL INFORMATION

26. What is your sex?

- ○ Male
- ○ Female
- ○ Other (please specify)

27. What is your marital status?

- ○ Married or common law
- ○ Divorced, widowed or separated
- ○ Single, never married

28. What is your age in years?

[ ]

29. What are the first three digits of your Postal Code? (used only to assess health region)

[ ]

30. What is your annual household income?

- ◯ < $20, 000
- ◯ $20, 000 - $40, 0000
- ◯ $40, 000 - $60, 000
- ◯ > $60, 000

31. What is the highest level of education you have completed?

- ◯ Less than high school
- ◯ High school diploma
- ◯ Trade school or college diploma
- ◯ University, undergraduate degree
- ◯ University, graduate degree

32. Have you ever experienced a side effect from a drug you were**prescribed**?

- ◯ Yes
- ◯ No

DRUG REACTIONS

33. Would you say the side effect was:

- ◯ Mild
- ◯ Moderate
- ◯ Severe

Public opinion survey about whole genome sequencing

FAMILY HISTORY

34. Have you or anyone in your family ever been diagnosed with a genetic condition?

- ◯ Yes
- ◯ No
- ◯ Unsure

35. Have you ever used services from direct to consumer genetic testing companies such as 23 and me or Ancestry.com?

- ◯ Yes
- ◯ No

36. Were you aware that Newfoundland and Labrador has a Provincial Health Research Ethics Board that oversees all health research in the province?

- ◯ Yes
- ◯ No

37. Have you ever had genetic counseling?

- ◯ Yes
- ◯ No

Public opinion survey about whole genome sequencing

FINAL THOUGHTS

38. Is there anything else you would like to share about whole genome sequencing? Please feel free to note anything you think is important, but not asked about in this survey.

Public opinion survey about whole genome sequencing

THANK YOU

That is the end of our survey.

We sincerely thank you for taking time to give us your opinion and invite you to enter into a

prize draw for a $100 gift card.

The following link takes you to the prize draw entry and is not linked to your survey or responses.

Please click

(Clicking here will also submit your survey)

If not, please click "Done"

Thank you for taking the time to complete this survey!
